# Peptide–Peptide Co-Assembly: A Design Strategy for Functional Detection of C-peptide, A Biomarker of Diabetic Neuropathy

**DOI:** 10.3390/ijms21249671

**Published:** 2020-12-18

**Authors:** Kiat Hwa Chan, Jaehong Lim, Joo Eun Jee, Jia Hui Aw, Su Seong Lee

**Affiliations:** 1 Division of Science, Yale-NUS College, 16 College Avenue West, Singapore 138527, Singapore; jiahui.aw@yale-nus.edu.sg; 2 Institute of Bioengineering and Nanotechnology, 31 Biopolis Way, The Nanos, Singapore 138669, Singapore; jaehongl@gmail.com (J.L.); jooeunjee@gmail.com (J.E.J.)

**Keywords:** peptide-peptide, co-assembly, design, detection, C-peptide, biomarker, diabetic neuropathy, inhibition, amyloidosis

## Abstract

Diabetes-related neuropathy is a debilitating condition that may be averted if it can be detected early. One possible way this can be achieved at low cost is to utilise peptides to detect C-peptide, a biomarker of diabetic neuropathy. This depends on peptide-peptide co-assembly, which is currently in a nascent stage of intense study. Instead, we propose a bead-based triple-overlay combinatorial strategy that can preserve inter-residue information during the screening process for a suitable complementary peptide to co-assemble with C-peptide. The screening process commenced with a pentapeptide general library, which revealed histidine to be an essential residue. Further screening with seven tetrapeptide focused libraries led to a table of self-consistent peptide sequences that included tryptophan and lysine at high frequencies. Three complementary nonapeptides (9mer com-peptides), wpkkhfwgq (Trp-D), kwkkhfwgq (Lys-D), and KWKKHFWGQ (Lys-L) (as a negative control) were picked from this table for co-assembly studies with C-peptide. Attenuated total reflectance Fourier transform infrared (ATR-FTIR) and circular dichroism (CD) spectroscopies were utilized to study inter-peptide interactions and changes in secondary structures respectively. ATR-FTIR studies showed that there is indeed inter-peptide interaction between C-peptide and the tryptophan residues of the 9mer com-peptides. CD studies of unaggregated and colloidal C-peptide with the 9mer com-peptides suggest that the extent of co-assembly of C-peptide with Trp-D is greatest, followed by Lys-D and Lys-L. These results are promising and indicate that the presented strategy is viable for designing and evaluating longer complementary peptides, as well as complementary peptides for co-assembly with other polypeptides of interest and importance. We discuss the possibility of designing complementary peptides to inhibit toxic amyloidosis with this approach.

## 1. Introduction

Proinsulin connecting peptide, in short C-peptide, is a 31-residue peptide that links the A and B chains of proinsulin. Upon enzymatic cleavage of C-peptide from pro-insulin in the pancreas, C-peptide is released into the blood stream along with insulin (A- and B-chains bonded together) in equimolar quantities. Early on, C-peptide was considered to be a side-product with insignificant bioactivity, but it was later discovered to influence a wide variety of physiological complications linked to diabetes, such as neuropathy, nephropathy, and encephalopathy [1]. Diabetic neuropathy is a debilitating condition in which the patient gradually loses sensation in the limbs due to progressive death of peripheral nerve cells. The loss of sensation makes the patient more susceptible to injuries, which ultimately could lead to amputation of the afflicted limb. It has been shown that the timely administration of C-peptide is able to ameliorate and reverse the degrading effects of neuropathy [2,3]. Just as APL1b28 [4] and natriuretic peptides [5,6] can serve as biomarkers for Alzheimer’s disease and myocardial ischemia reperfusion injury respectively, it is desirable to monitor C-peptide concentration in the blood to permit timely intervention [7,8].

In order to attain early diagnosis of diabetic neuropathy, it is imperative to have a simple method of detection. Mass spectrometry (MS)-based methods have been developed for the detection of peptide biomarkers. For instance, matrix-assisted laser desorption/ionization-time-of-flight (MALDI-TOF) MS has been used to differentiate between community-associated methicillin-resistant *Staphylococcus aureus* (MRSA), hospital-associated MRSA and vancomycin intermediate SA via peptide biomarkers [9], as well as tracking multiple peptides using selective reaction monitoring [10]. In fact, an automated protocol for analyzing serum peptides has also been developed [11]. However, mass spectrometry requires bulky instrumentation and thus is not readily usable at home. Antibodies for C-peptide have been developed and are already commercially available in diagnostic kits. However, each diagnostic test can cost up to a few US dollars even in India (according to Medifee.com), which can make more regular testing considerably costly for low income families. Given that the production of antibodies is relatively more expensive compared to peptide synthesis, it has been proposed that peptides can be an attractive alternative to antibodies due to the lower cost of production and ability to mimic protein surfaces [12].

The design of complementary peptides that can interact specifically, or co-assemble, with another peptide, however, has not been attempted. Peptide-peptide co-assembly, currently in a nascent stage of intense study, certainly possesses great potential for building complex multifunctional nanostructures [13]. Although peptide-peptide co-assembly is uncommon in nature, it has been demonstrated to be possible [14] and has already been utilized to construct microgels for bionanotechnological applications [15]. However, it is not trivial to design a complementary peptide de novo, since the rules governing peptide-peptide interaction are not well-established yet. Recently, Bera et al. reported the ability of various amino acids to self-assemble and this represents a first important step towards eventually establishing universal rules for designing a complementary peptide to a target peptide sequence [16]. For now, however, an alternative strategy is needed to design a complementary peptide to C-peptide.

The utility of peptides as a protein capture agent has been a fruitful enterprise [17]. Based on a bead-based combinatorial approach, peptide-based protein capture agents have already been developed for proteins such as carbonic anhydrase isozymes [18], prostate-specific antigen [19], kinases (important protein targets for various disease states) [20], fluorescent chemosensors for Ser/Thr kinases [21], and the Akt inhibitor [22]. Such a templated approach has also been used to uncover modulators of protein/protein interactions [23]. Given the success of the bead-based approach to discover protein capture agents, we envisage using the same approach to search and locate a peptide capture agent for C-peptide. It has been reported before that the heptapeptide LVEALYL, a segment in insulin B chain, is able to induce the aggregation of insulin [24]. This suggests that LVEALYL may be able to co-assemble with insulin to effectuate aggregation of the latter. Thus, it is conceivable that a complementary peptide that can co-assemble with C-peptide can be discovered through a bead-screening process.

## 2. Results and Discussion

### 2.1. Bead Screening Strategy for Complementary Peptide to C-Peptide

The bead screening search process commenced with a “5-mer” general library in which each bead contains a D-amino acid-based pentapeptide attached to its surface (Ac-x_1×2_x_3_x_4_x_5_-gaba-gaba-r-m-bead). D-amino acids are used in order to resist degradation by serum proteases in blood. A total of 17 D-amino acids (excluding cysteine, methionine, and arginine) are utilised. γ-aminobutyric acid (GABA) acts to increase the length between the 5-mer sequence and the Tentagel bead. Cysteine is removed from the library due to its vulnerability of oxidation. Methionine is excluded as it is present in all beads to facilitate cleavage of the peptide from the bead with cyanogen bromide [25]. Arginine is also present on all the beads to facilitate identification of the peptide sequence during MALDI analysis [26]. One copy of all 1.4 × 10^6^ (17^5^) members of a 5-mer library requires about 0.5 g of Tentagel resin (2.86 × 10^6^ beads/g). With the COPAS bead sorter system, 100 mg of resin can be efficiently sorted each time. Thus, screening a 5-mer general library four times will enable us to cover ~80 % of the permutational space, which is substantial and efficiently achieved. Contrast this to the screening of 7-mer and 9-mer random libraries: one copy of all possible permutations will respectively be 4.1 × 10^8^ (17^7^) and 1.2 × 10^11^ (17^9^), which in turn would respectively require 143 g and 41 kg of Tentagel beads! It is clearly impossible to screen a substantial portion of the permutational space of larger general libraries.

**Figure 1:**
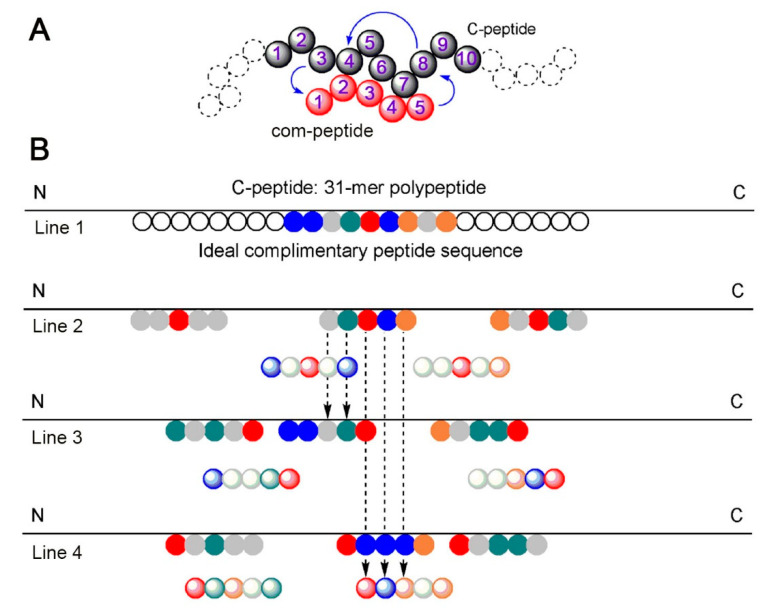
The various coloured circles represent different amino acid residues. The dotted arrows point to the segments of pentapeptide sequences that can be picked up a focussed library possessing those segments. (**A**) Illustration of how inter-peptide interaction is also affected by intra-peptide residue interaction. In this example, residue 3 of C-peptide may interact favourably with residue 1 of the com-peptide. This would affect how residues 2–5 of the com-peptide interact with residues 4–8 of the C-peptide. (**B**) Illustration of the triple-overlay strategy for the search of a complementary peptide (com-peptide) to co-assemble with C-peptide. The pentapeptide in Line 2 can pick up two further pentapeptide sequences in Lines 3 and 4 via the consensus triplet sectors.

C-peptide is a 31-amino acid polypeptide that largely adopts a random coil structure in solution [27]. As such, the complementary peptide (com-peptide) that can co-assemble with C-peptide is likely to be permutationally flexible, in which the residues of com-peptide are inter-dependent on each other to interact optimally with C-peptide. For example, the identities of amino acid residues 1–5 will affect the optimal identities of residues 3–7 since every residue has an optimal twist and turn that must be compromised in the co-assembly of com-peptide with C-peptide (Figure 1A). This point is manifest by the big influence a small molecular change exerts on the self-assembly of peptides [28]. Thus, the search for a suitable com-peptide has to preserve inter-residue information during the process. In order to achieve this, we devise and utilise a triple-overlap combinatorial strategy to preserve inter-residue information during our search for a suitable com-peptide. We utilize the fact that effective peptide-peptide interaction only requires a minimum of three amino acid residues to mediate, as in the case of protein-folding [29]. Figure 1B illustrates how this triple-overlap strategy works. The first line depicts a hypothetical com-peptide with its sequence represented by various coloured circles. The start of the search may yield a certain pentapeptide as depicted on Line 2. The first three residues may serve as the consensus triplet sector for two more searches that would yield the sequences in Lines 3 and 4. All the residues in Lines 2–4 combined would yield an ideal 9-mer sequence in com-peptide as depicted in Line 1. In this way, one may systematically comb through the vast permutational space while preserving inter-residue information throughout the search process. This strategy is analogous to peptide and nucleotide sequencing in mass spectrometry, in which fragments with the same consensus sequences are aligned together to reconstruct the original sequence.

**Figure 2:**
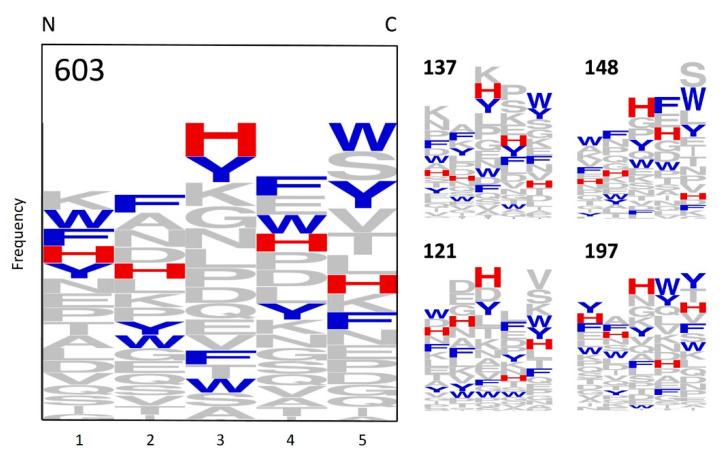
Summary of the bead screening results of a 5mer general library against C-peptide. Histidine, the aromatic residues (phenylalanine, tyrosine, and tryptophan), and the remaining residues are respectively highlighted in red, blue, and grey. The numbers indicate the number of peptide sequences successfully sequenced. The histogram on the left represents the composite results of the four separate rounds of screening (on the right) with the 5mer general library. The histograms are prepared with the publicly available WebLogo program [30]. These results show that it is consistently favourable for His to be in position 3.

Thus, C-peptide was incubated with 100 mg of the 5-mer general library four times, which corresponds to ~80% of the permutational space as described earlier. The outcome of the four rounds of screening is summarized in Figure 2. The relative heights of the letters in each column represent the frequencies at which the various amino acids appeared in each position of the analysed 5-mer sequences. Evidently, His (in red) consistently appeared with the highest frequencies in position 3, suggesting that His promotes co-assembly of the pentapeptide with C-peptide. Thus, three focused libraries with His as the anchor residue in the first, third, and fifth positions were designed and prepared. Being “4-mer” focused libraries, there are 8–9 copies of each sequence in every 100 mg of Tentagel resin. Since the 4-mer focused libraries are small enough, it is viable to screen each focused library three times against C-peptide. The high prevalence of Phe, Tyr, and Trp in the general library search (Figure 2) also prompted us to investigate to design the following three Phe/Tyr/Trp-centric focused libraries: Ac-x-4mer (x = w, y, h, f, r), Ac-2mer-x-2mer (x = w, y, h, f, l), and Ac-4mer-x (x = w, y, h, v, s).

**Figure 3:**
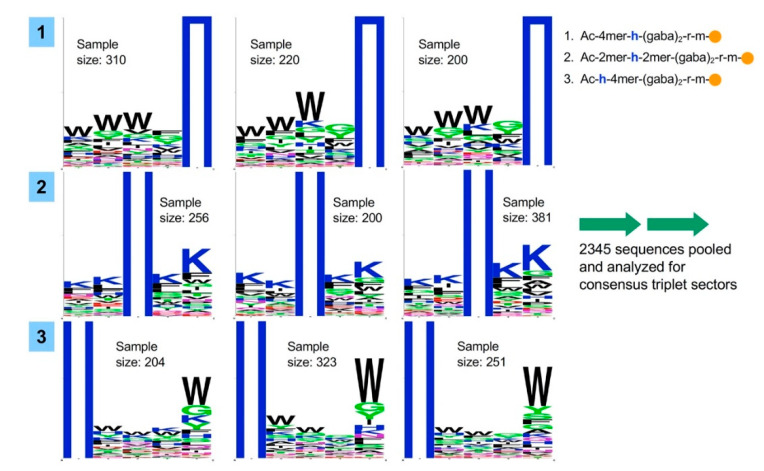
Summary of the screening results of the three His-centric focused libraries against C-peptide (panels 1–3). The twin blue bars in positions 5 (row 1), 3 (row 2), and 1 (row 3) correspond to His. The results of row 1 indicate that it is favourable for Trp to be in positions 1–3 when His is in position 5. The results of row 2 indicate that it is favourable for Lys to be in positions 4 and 5 when His is in position 3. The results of row 3 indicate that it is strongly favourable for Trp to be in positions 5 when His is in position 1.

**Figure 4:**
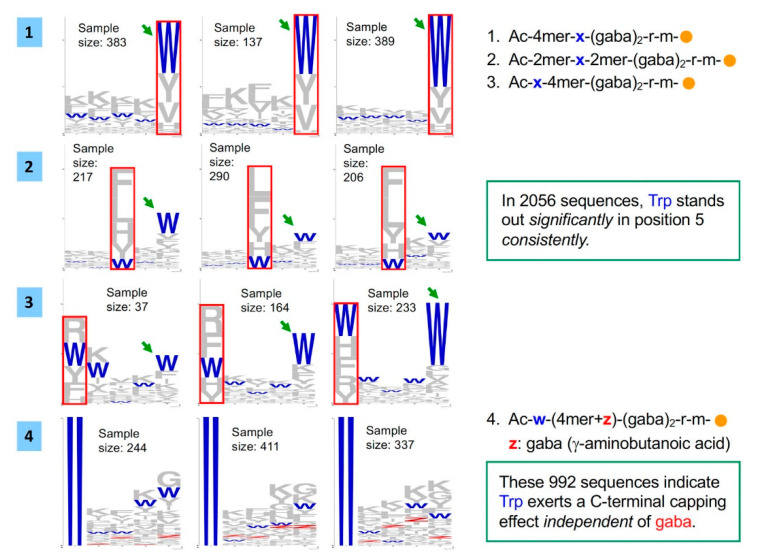
Summary of the screening results of the four Phe/Tyr/Trp-centric focused libraries against C-peptide (panels 1–4). The red boxes correspond to the position of the focused library in which variation in residues is limited. The green arrows highlight the high frequency at which Trp appeared in the peptide sequences that interacted favourably with C-peptide during the screening process.

The results of the search with the three His-centric “4-mer” focused libraries are presented in Figure 3. Each row presents the triplicate results for each His-centric focused library. It is evident that each set of triplicate results is largely in agreement with each other: in row 1, when His is in position 5, Trp is consistently represented with high frequency in positions 1–3 whereas in row 3, Trp is represented strongly only in position 5 when His is in position 1. On the other hand, when His is in position 3 (row 2), Lys is represented more frequently, in particular in position 5. For the Phe/Tyr/Trp-centric focused libraries, Figure 4 shows that Trp consistently dominates position 5. As the most consistent aspect of these Phe/Tyr/Trp-centric focused libraries is the presence of GABA after position 5, we sought to assess the importance of GABA. We designed a focused library (with Trp in position 1) in which GABA was included in positions 2–5. If GABA were to augment the interaction of Trp with C-peptide, it would stand out prominently in either position 2, 3, or both (these are the relative positions of GABA to Trp in the Phe/Tyr/Trp-centric focused libraries). However, Figure 4 shows that the frequency of GABA is consistently low, indicating that Trp exerts its C-terminal capping effect independently of GABA.

**Figure 5:**
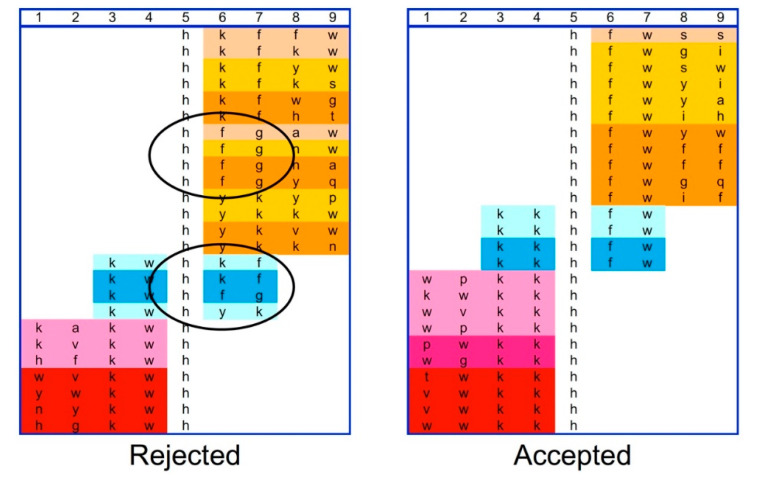
Illustration of the process of rejecting and accepting complementary peptide sequences to C-peptide. In the left table, the pink and red libraries, as well as the light and dark blue libraries, possess kw (positions 3 and 4) in each coloured library and in two different rounds (e.g., pink and red), so they fulfil the two criteria for acceptance. However, positions 6 and 7 fail one or both criteria (lower oval). For example, kf may appear in two different rounds (Criterion 1), but it only appears once in each round. Both fg and yk only appear once in either round. Thus, even though fg fulfils both criteria in the orange/dark yellow/cream library (upper oval), this whole table is rejected because positions 1–5 cannot link up with positions 5–9. On the other hand, all sectors in the right table fulfil both criteria of acceptance, so the self-consistent table on the right is accepted.

Given that triplicate results for each His-centric focused library search are consistent with each other, it is possible to compile and analyse them for consensus triplet sectors. This was achieved by aligning all the sequences with respect to His as illustrated in Figure 5; the different colours reflect various triplicate rounds for each library. We apply the criteria that the consensus sectors on overlapping libraries have to (1) manifest at least two out of three rounds and (2) at least twice in those respective rounds. Positions 3–5 in this table, which are reflected by the two different shades of blue for consensus sector kwh, meet these two criteria. However, positions 5–7 do not meet criterion 2. Thus, this set was disqualified along with rejection of the entire table. On the other hand, all three focused libraries in the table on the right fulfils both criteria simultaneously, so this group of self-consistent sequences are accepted. Gratifyingly, this table of sequences also has Trp in “position 5” of the central segment (positions 3–7), which agrees with the results of the Phe/Tyr/Trp-centric focused libraries. Here, one can see how the three focused libraries can counter-check one another. This counter-check also possess the added advantage of reducing the impact of unequal data set sizes among triplicates (which is inevitable as sequences may be lost during the peptide cleavage process or peptides cannot be properly sequenced due to poor quality of the mass spectra) as it is the overlap with related focused libraries that one is looking for.

The search results in Figure 5 suggest that the screening process is successful. Five acidic residues, i.e., four glutamic acid and one aspartic acid residues (underlined), are present in C-peptide (EAEDLQVGQVELGGGPGAGSLQPLALEGSLQ), so it can be expected that a complementary peptide (com-peptide) should possess a large number of basic residues. This is indeed what we observe, with a high prevalence of Lys and His residues. As mentioned at the end of Introduction, since the heptapeptide LVEALYL is presumably capable of co-assembling with insulin, the nonapeptide sequences in Figure 5 should also be of sufficient length to co-assemble with C-peptide. Thus, two nonapeptide sequences (9mer com-peptides) were picked to study their co-assembly with C-peptide. They are wpkkhfwgq (Trp-D) and kwkkhfwgq (Lys-D). For positions 1–5, wpkkh was picked as it appeared twice in the final table. kwkkh was the other sequence has it possesses one more Lys (and hence one more positive charge) compared to wpkkh and all other alternatives, so one can assess the effect of an additional basic residue. For positions 5–9, hfwgq was picked as the alternatives contain more aromatic residues, which might promote self-assembly of the 9mer com-peptide. KWKKHFWGQ (Lys-L), which is the L-enantiomer of Lys-D was also assessed as a negative control. The N- and C-termini of the 9mer com-peptides are underivatized, i.e., they are -NH_2_ and CO_2_H respectively.

### 2.2. Spectroscopic Study of C-Peptide–Com-Peptide Coassembly

The co-assembly of the com-peptide and C-peptide was studied by attenuated total reflectance Fourier transform infrared (ATR-FTIR) spectroscopy and circular dichroism (CD spectroscopy) [31,32]. ATR-FTIR spectroscopy can potentially provide information on inter-peptide interaction, especially since we can directly analyse the samples in solution. To do this, we compared the IR spectra of the 9mer com-peptides and C-peptide in phosphate buffer with the theoretical spectra of a mixture in which the 9mer com-peptides and C-peptide do not interact. To derive the theoretical spectra of two equimolar non-interacting species from the spectra of the two species,

(1) $$T=\;\frac{I_{o}-I}{I_{o}}$$

(2) $$T_{a+b}=\;\frac{I_{o}-I_{a}-I_{b}}{I_{o}}$$

(3) $$T_{a+b}=1-\;\frac{I_{a}}{I_{o}}-\frac{I_{b}}{I_{o}}$$

(4) $$T_{a+b}=1-\left(1-T_{a}\right)-\left(1-T_{b}\right)$$

Therefore,

(5) $$T_{a+b}=T_{a}+T_{b}-1$$

**Figure 6:**
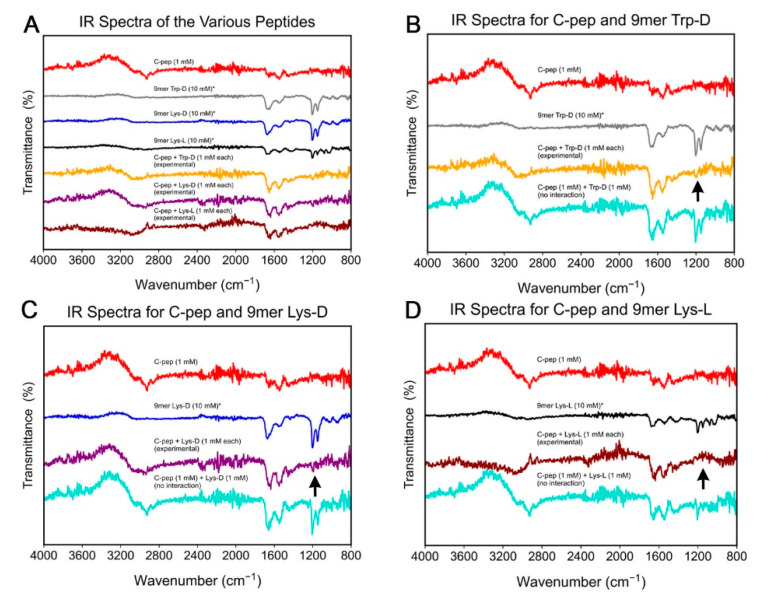
ATR-FTIR spectra of C-peptide, Trp-D, Lys-D, and Lys-L. Panel (**A**) compares and contrasts the IR spectra of C-peptide, 9mer com-peptides, and the mixtures. Panels (**B**–**D**) compare the experimental IR spectra of C-peptide+com-peptide with the theoretical spectrum, C-peptide and the respective com-peptide. The arrows in spectra (**B**–**D**) indicate the main difference between the experimental and theoretical spectra. The disappearance of the peaks around 1200 cm^−1^ suggests that there is interaction of Trp with C-peptide during co-assembly. The bump around 3300 cm^−1^ is due to imperfect subtraction of the background water stretching mode.

Or,

(6) $$T_{a+b}\left(\%\right)=T_{a}\left(\%\right)+T_{b}\left(\%\right)-100$$

where *T* is transmittance, *I_o_* is the incident light, *I* is the light absorbed by the species. As the derivation shows, the theoretical transmittance (%) spectrum can be simply obtained according to Equation (1). The various spectra are plotted and stacked with the relevant spectra in Figure 6. As the spectra illustrates, the most prominent difference as a result of co-assembly lie around 1200 cm^−1^, in which the two peaks in that region due to the 9mer com-peptide are absent in the 9mer C-peptide+com-peptide spectra. These two peaks are attributable to the amide III vibrational mode of Trp [33,34]. Thus, the disappearance of these two peaks in the co-assembly mixture indeed suggests that there is interaction between the Trp of the 9mer com-peptide and C-peptide during co-assembly, which may be related to the ability of tryptophan to stabilize β-hairpin peptides via hydrophobic interactions [35]. This is congruous with the bead screening results, in which Trp was found to be highly favoured (panels 1 and 3 of Figure 3).

**Figure 7:**
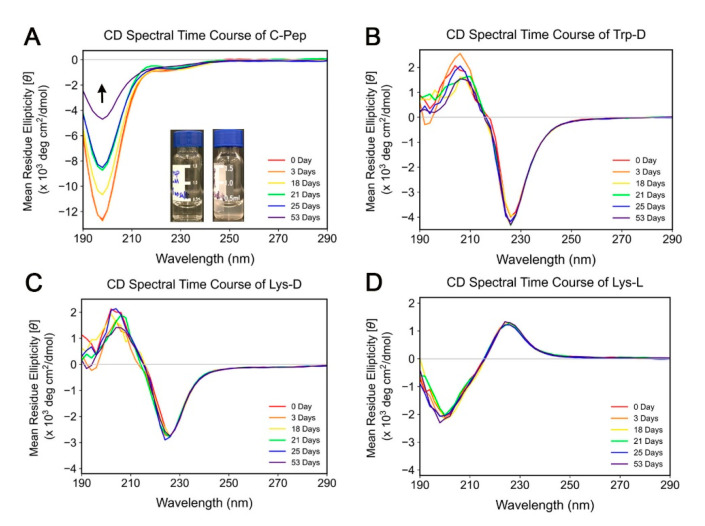
CD spectral time course of (**A**) C-peptide, (**B**) Trp-D, (**C**) Lys-D, and (**D**) Lys-L. The inset in spectrum A shows the change in C-peptide over time–(left) clear at the start, (right) colloidal after 53 days. The CD spectra show that C-peptide changes slowly over time, but the three 9mer com-peptides remain essentially unchanged over the same period.

Complementary to ATR-FTIR spectroscopy, CD spectroscopy is able to provide information on changes in the secondary structures after C-peptide–com-peptide co-assembly [31,32,36]. Figure 7 illustrates the CD spectra of C-peptide, Trp-D, Lys-D, and Lys-L. The CD spectra of C-peptide suggest that it largely adopt a polyproline II helical structure, as the negative peaks at 198 nm and 226 nm suggest [37,38]. On the other hand, the CD spectra of Trp-D and Lys-D possess spectral features of an antiparallel β-sheet structure (positive peak ~198 nm and negative peak at ~225 nm) [39]. Lys-L, being the enantiomer of Lys-D, possesses spectral features with opposite phases to Lys-D. However, the absolute intensities of the peaks at the same wavelength differ, suggesting that the enantiomers do not simply self-assemble into supramolecular structures with opposite chiralities [40], but differ a little in some structural details; this aspect is not explored further in this study. All the peptides were also monitored over time. While Trp-D, Lys-D, and Lys-L essentially remained unchanged over 53 days, C-peptide was observed to aggregate slowly as observed previously [41]–the negative peak at 198 nm decreased in intensity gradually and the initial clear solution became colloidal over time (inset of Figure 7A).

As with the IR spectra, we compared the CD spectra of the 9mer com-peptides and C-peptide in phosphate buffer with the theoretical spectra of a mixture in which the 9mer com-peptides and C-peptide do not interact. To derive the theoretical spectra of two equimolar non-interacting species from the spectra of the two species,

(7) $$\theta_{t}=\;\theta_{C}+\;\theta_{n}$$

(8) $$[\theta_{t}]c_{t}r_{t}l=\;[\theta_{C}]c_{C}r_{C}l+\left[\theta_{n}\right]c_{n}r_{n}l$$

(9) $$c_{t}r_{t}=\;c_{C}r_{C}+\;c_{n}r_{n}$$

(10) $$c_{t}r_{t}=\left(1\times 31\right)+\left(1\times 9\right)$$

(11) $$c_{t}r_{t}=\;40$$

**Figure 8:**
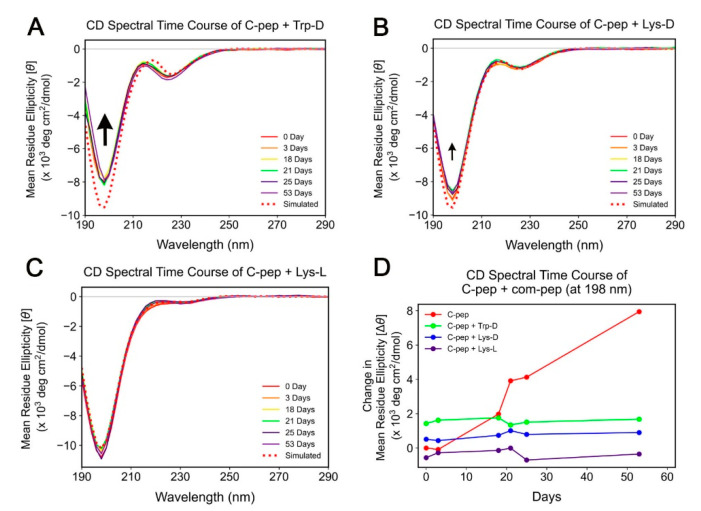
CD spectral time course of unaggregated C-peptide co-assembled with (**A**) Trp-D, (**B**) Lys-D, and (**C**) Lys-L respectively. The relative sizes of the vertical arrows denote the relative change in ellipticity at 198 nm. Panel (**D**) illustrates the change in the mean residue ellipticity at 198 nm relative to the simulated spectra of non-interaction between C-peptide and the 9mer com-peptides; the line due to C-peptide reflects changes relative to 0 day. It shows that Trp-D interacts to the greatest extent with C-peptide, followed by Lys-D and (minimally) Lys-L. It also shows how the electrolyte-like 9mer com-peptides stopped the aggregation of C-peptide.

Therefore,

(12) $$[\theta_{t}]\times 40=\;[\theta_{C}]\left(1\times 31\right)+[\theta_{n}]\left(1\times 9\right)$$

(13) $$simulated\;\left[\theta_{t}\right]=\;\frac{31\left[\theta_{C}]+9[\theta_{n}\right]}{40}\;\times \;10^{3}\;\deg\cdot {\mathrm{cm}}^{2}/\mathrm{dmol}$$

where θ, [θ], *c*, *r* and *l* are the ellipticity (mdeg), mean residue ellipticity (deg cm^2^/dmol), concentration (in mM; 1 mM), number of residues, and cuvette pathlength (in mm). The lengths of C-peptide and the 9mer com-peptide are 31 and 9 respectively. As Figure 8 illustrates, co-assembly of C-peptide with either Trp-D or Lys-D leads to a significant change in the CD spectra. Gratifying, no such change was observed when Lys-L, the negative control, was mixed with C-peptide. This continues to be the case even when the mixture was monitored for 53 days, i.e., no change occurred. This indicates that the experimental spectra of C-pep+Lys-L were simply a non-interacting equimolar mixture of C-peptide and Lys-L. This suggests that there is indeed some degree of specificity in interaction of the D-enantiomer, Lys-D, with C-peptide. However, if C-peptide and Lys-L were truly non-interacting, then the spectrum due to C-peptide should continue to evolve as Figure 7A and Figure 8D. One possible explanation is that the positively charged 9mer com-peptides function as an electrolyte to stabilize the negatively-charged C-peptide and avert aggregation [42]. In addition, given that the magnitude of change induced by Trp-D is greater than Lys-D (Figure 8D), one is tempted to suggest that extent of co-assembly of C-peptide with Trp-D is greater than with Lys-D.

**Figure 9:**
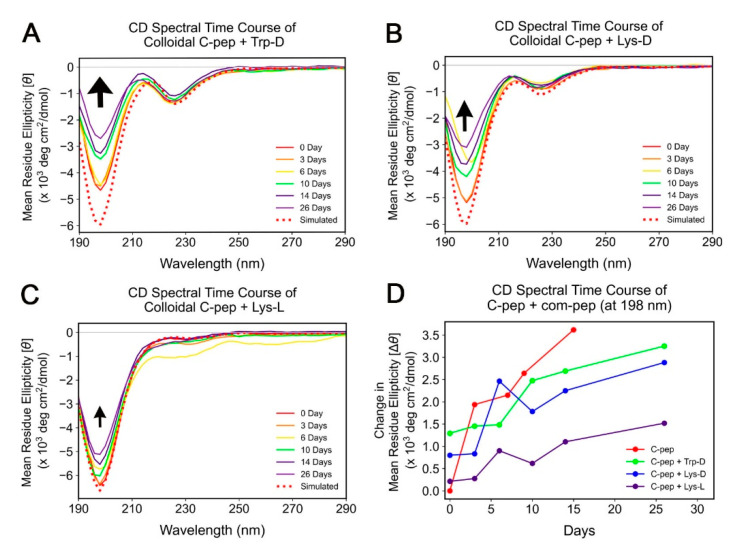
CD spectral time course of colloidal C-peptide co-assembled with (**A**) Trp-D, (**B**) Lys-D, and (**C**) Lys-L respectively. The relative sizes of the vertical arrows denote the relative change in ellipticity at 198 nm. Panel (**D**) illustrates the change in the mean residue ellipticity at 198 nm relative to the simulated spectra of non-interaction between colloidal C-peptide and the 9mer com-peptides; the line due to C-peptide reflects changes relative to 0 day. It shows that Lys-L interacts to the greatest extent with colloidal C-peptide (biggest decrease in rate of increase of [θ]_198_ relative to that of C-peptide), followed by Lys-D and Trp-D.

We also sought to study the effect of the 9-mer com-peptides on colloidal C-peptide as a way to assess the extent of C-peptide–com-peptide co-assembly. The hypothesis is that if the co-assembly of Trp-D or Lys-D with C-peptide were strong enough, the aggregation of C-peptide would be reversed. Physically, the C-peptide colloid would become clear again. Spectroscopically, [θ]_198_ would decrease. However, these observations were not made. As Figure 9D shows, [θ]_198_ continues to increase, albeit at a lower rate in the presence of the 9mer com-peptides. In addition, the rate of increase of [θ]_198_ is: Trp-D ≈ Lys-D ≫ Lys-L. This may reflect the low concentration of unaggregated C-peptide and how Trp-D and Lys-D might be specific in their interaction with unaggregated C-peptide whereas Lys-L is able to interact more efficiently with colloidal C-peptide instead. This also suggests that the co-assembly of Trp-D or Lys-D with C-peptide is not strong enough to reverse the aggregation of C-peptide. 

Last but not least, we sought to establish if there is a well-defined binding ratio between C-peptide and the 9mer com-peptides. This was accomplished by the continuous variation method, i.e., the plotting of a Job plot, as has been carried out by Truex and Nowick for their peptide co-assembly system [43]. In this method, the total concentration of C-peptide and 9mer com-peptide was fixed, at 1 mM. The relative concentration of C-peptide to com-peptide was varied in fixed steps of 0.1 mM, viz. from 0 mM C-peptide/1 mM com-peptide to 0.1 mM C-peptide/0.9 mM com-peptide, and finally to 1 mM C-peptide/0 mM com-peptide. There needs to be a distinct signal characteristic of the co-assembled C-peptide–com-peptide. For this, we utilise the CD signal. As Figure 8 shows, there are distinct signals for all the peptides at 198 nm; the biggest change in the CD signals also occurs at 198 nm. Thus, the co-assembled C-peptide/com-peptide will have a distinct CD signal at 198 nm too. By comparing the experimental signal with the theoretical signal (of assumed non-interaction between the two species), one would be able to derive the change in ellipticity at 198 nm due to the co-assembled C-peptide–com-peptide. Thus,

(14) θ =[θ]crl

(15) $$c_{t}r_{t}=\;c_{C}r_{C}+\;c_{n}r_{n}$$

(16) $$c_{t}r_{t}=\;\left(x\times 31\right)+\left[\left(1-x\right)\times 9\right]$$

(17) $$c_{t}r_{t}=\;22x+9$$

Therefore,

(18) $$experimental\;\left[\theta_{t}\right]=\frac{10\;\theta_{t}}{22x+9}\;\times \;10^{3}\;\deg\cdot {\mathrm{cm}}^{2}/\mathrm{dmol}$$

(19) $$\theta_{t}=\;\theta_{C}+\;\theta_{n}$$

(20) $$[\theta_{t}]c_{t}r_{t}l=\;[\theta_{C}]c_{C}r_{C}l+\left[\theta_{n}\right]c_{n}r_{n}l$$

From Equation (17),

(21) $$[\theta_{t}]\left(22x+9\right)=\;[\theta_{C}]\left(x\;\times \;31\right)+[\theta_{n}]\left[\left(1-\;x\right)\times 9\right]$$

Therefore,

(22) $$simulated\;\left[\theta_{t}\right]=\;\frac{31x\left[\theta_{C}]+9\left(1-x\right)[\theta_{n}\right]}{22x+9}\;\times \;10^{3}\;\deg\cdot {\mathrm{cm}}^{2}/\mathrm{dmol}$$

Finally,

(23) $$\mathrm{peptide}-\mathrm{peptide}\;\mathrm{co}-\mathrm{assembly}\left[\theta_{t}\right]=experimental\left[\theta_{t}\right]-simulated\left[\theta_{t}\right]$$

**Figure 10:**
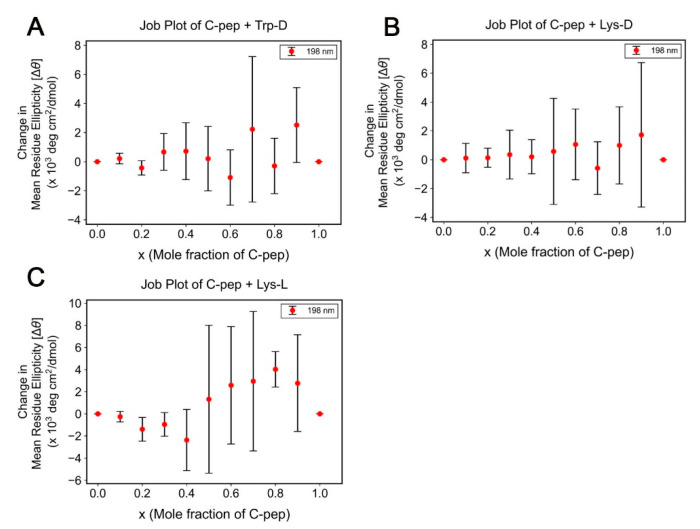
Job plots of C-peptide and (**A**) Trp-D, (**B**) Lys-D, and (**C**) Lys-L at a total concentration of 1 mM as determined at 198 nm. The standard deviations are derived from three separate measurements over the course of two weeks. The absence of a well-defined peak in the plots suggests that the co-assembly of C-peptide and the 9mer com-peptides is not optimized yet.

The various values of C-peptide–com-peptide co-assembly [$\theta_{t}]$_198_ are then plotted against *x* (mole fraction of C-peptide). The presence of a sharp peak in the Job plot would be indicative of a well-defined C-peptide–com-peptide co-assembled complex with a ratio defined by *x*/(1 − *x*), where *x* is the position of the peak. As Figure 10 shows, there is not a well-defined peak for any C-peptide–com-peptide co-assembly structures formed. The standard deviations, which reflect the state of the peptide-peptide co-assembly over two weeks, also suggest that there was considerable annealing of the co-assembled structures over time, leading to different CD absorptions. Similarly, attempts to fit binding models to data collected from isothermal titration calorimetry (ITC) [44] or microscale thermophoresis (MST) [45] studies of C-peptide and the 9mer com-peptides were unsuccessful. This is in contrast to the ITC results of Bera et al., who were able to measure dissociation constants (K_d_) in the micromolar range even for single amino acid-single amino acid interactions [16]. These observations suggest that the co-assembly of C-peptide with either Trp-D or Lys-D has not been optimized, so that there are many different ways C-peptide can interact with either Trp-D or Lys-D (i.e., no fixed binding ratio).

## 3. Materials and Method

**Scheme 1:**
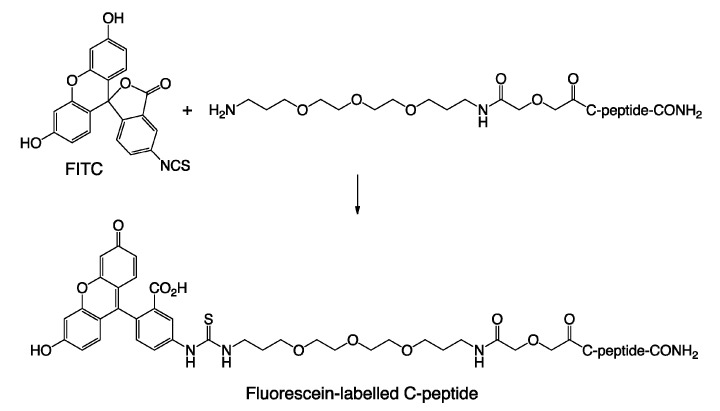
Reaction for the labeling of C-peptide for visualization via fluorescence during the bead sorting process.

**Scheme 2:**
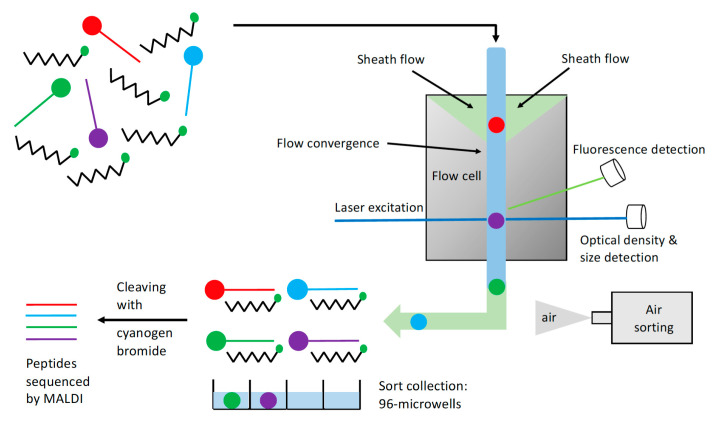
Illustration of the bead screening process for a complementary peptide that can co-assemble with C-peptide. The process begins with incubating fluorescein-labelled C-peptide with the bead library. Sequences that can co-assemble with C-peptide will fluoresce on excitation with a blue laser, which will be sorted and collected in a 96-microwell. The collected beads are then treated with cyanogen bromide to cleave the peptides off the beads. Finally, the recovered peptides are analysed by matrix-assisted laser desorption/ionization mass spectrometry (MALDI-MS) and sequenced by the PEAKS mass spectrometry program.

The following chemicals were purchased from the respective companies: Fmoc-Rink amide-MBHA resin, natural and non-natural Fmoc-protected amino acids (GL Biochem (Shanghai) Ltd., Shanghai, China); TentaGel S amino resin (AnaSpec, Fremont, CA, USA); α-cyano-4-hydroxycinnamic acid (Bruker, Billerica, MA, USA); *N*-methylpyrrolidone, diethyl ether, dichloromethane (Merck, Kenilworth, NJ, USA); fluorescein isothiocyanate (Sigma-Aldrich, St. Louis, MO, USA). C-peptide that was used during the bead screening process was prepared via solid-phase peptide synthesis by following the sequence (i.e., EAEDLQVGQVELGGGPGAGSLQPLALEGSLQ) as reported before [27]. In addition, C-peptide is labelled with fluorescein (Scheme 1) so that beads that co-assemble with C-peptide may be visualized by fluorescence. Given that fluorescein is attached far away from C-peptide, the fluorescence property of fluorescein moiety is not expected to differ significantly from the native fluorescein isothiocyanate (FITC) [46]. C-peptide and the three nonapeptides utilized for co-assembly studies were purchased from Cellmano Biotech Limited (Hefei, China). The following procedures were carried out with the respective instruments: One-bead-one-compound (OBOC) peptide libraries and bulk peptide preparations (Titan 357 automatic synthesizer, AAPPTec); bead sorting (COPAS Plus, Union Biometrica); matrix-assisted laser desorption/ionization (MALDI) time-of-flight (TOF) mass spectrometry (MS) and MS/MS analyses (Bruker Autoflex II TOF/TOF spectrometer); microwave-assisted cleavage of peptides from beads (Sharp Model R-248); bulk peptide purification (Gilson preparative ultra performance liquid chromatography system equipped with a C_18_ reversed-phase preparative column – Kromasil, AkzoNobel; 5 mm, 250 × 30 mm). For each round of screening, 100 mg of OBOC peptide library is incubated in phosphate buffer (pH 7.4) with bovine serum albumin (as a blocking agent) overnight with constant shaking. The peptides recovered from the screening process were sequenced with the PEAKS mass spectrometry program. All these procedures have been described in detail previously and are represented in Scheme 2 [25,26,47]. The procedures for attenuated total reflectance Fourier transform infrared spectroscopy (ATR-FTIR) [48] and circular dichroism (CD) [49] spectroscopies have been previously reported by us.

## 4. Conclusion and Outlook

We have devised a simple and viable screening strategy of bead-based peptide libraries to search and locate possible 9mer sequences that can co-assemble with C-peptide. In this process, ATR-FTIR and CD spectroscopies have been found to be very useful in assessing the co-assembly interactions between C-peptide and the 9mer com-peptides. ATR-FTIR suggests that the Trp residues in the 9mer com-peptides interact significantly with C-peptide. CD suggests that C-peptide co-assembles most strongly with Trp-D, followed by Lys-D and Lys-L. On the other hand, the extent of co-assembly is reversed with colloidal C-peptide. CD-based Job plots also suggest that there is not a fixed binding ratio between C-peptide and any of the current com-peptides. All these experimental data suggest that the co-assembly between C-peptide and the current 9mer com-peptides is not optimal yet. Nonetheless, the strategy we have presented is sound for a continued search and evaluation that can lead to a more effective com-peptide for C-peptide. An avenue of improvement to improve the search process would be to utilise computational tools to refine and narrow the search for an ideal complementary peptide [50,51]. 

Besides designing a better complementary peptide for C-peptide, this approach can be employed for other polypeptides of interest and importance. One prominent candidate is the amyloid β-42 (Aβ_42_) polypeptide that has been implicated as the cause of Alzheimer’s disease and has been targeted for therapeutic invention [52]. Computationally assisted intensive efforts [53,54,55,56] have been undertaken by many researchers into designing therapeutic agents that can inhibit the folding of Aβ_42_ into toxic amyloid fibrils, but this has been met with varying degrees of success [57,58]. One of the reasons is that Aβ_42_ can adopt various conformations under various physiological conditions [59]. As a result, inhibitors that are designed to work under a certain specific condition may lose its efficacy under altered conditions. This is particularly pertinent for Alzheimer’s disease, which is a slow developing condition. In this regard, the bead-based approach presented here can be helpful. The screening process can be carried out various simulated physiological conditions to capture favourable binding sequences for various Aβ_42_ conformations. The screening results can then be compared and common fragment sequences can be assembled as we have described. Ideally, this would furnish a complementary peptide that can bind to Aβ_42_ under any conditions. Potentially, this would help to arrest Alzheimer’s disease at any stage of development by recognising and binding to various forms/conformations of Aβ_42_. This would certainly be complementary to current efforts and represent an advance in the ongoing quest to design an effective therapeutic agent for Alzheimer’s disease.
